# A Celecoxib Derivative Eradicates Antibiotic-Resistant *Staphylococcus aureus* and Biofilms by Targeting YidC2 Translocase

**DOI:** 10.3390/ijms21239312

**Published:** 2020-12-07

**Authors:** Shiou-Ru Tzeng, Yi-Wei Huang, Yao-Qing Zhang, Ching-Yi Yang, Han-Sheng Chien, Yi-Ru Chen, Sung-Liang Yu, Ching S. Chen, Hao-Chieh Chiu

**Affiliations:** 1 Institute of Biochemistry and Molecular Biology, College of Medicine, National Taiwan University, Taipei 10051, Taiwan; srtzeng@ntu.edu.tw (S.-R.T.); koichi199711@hotmail.com (Y.-Q.Z.); 2 Department of Clinical Laboratory Sciences and Medical Biotechnology, College of Medicine, National Taiwan University, Taipei 10048, Taiwan; r19817037@yahoo.com.tw (Y.-W.H.); popo305220@gmail.com (C.-Y.Y.); henry851013@gmail.com (H.-S.C.); rabiomix@gmail.com (Y.-R.C.); slyu@ntu.edu.tw (S.-L.Y.); 3 Department of Laboratory Medicine, National Taiwan University Hospital, College of Medicine, National Taiwan University, Taipei 10002, Taiwan; 4 Drug Development Center, China Medical University, Taichung 40402, Taiwan; cschencmu@gmail.com; 5 Department of Medical Research, China Medical University Hospital, China Medical University, Taichung 40447, Taiwan

**Keywords:** MRSA, translocon, thermal shift, surface plasmon resonance

## Abstract

The treatment of *Staphylococcus aureus* infections is impeded by the prevalence of MRSA and the formation of persisters and biofilms. Previously, we identified two celecoxib derivatives, Cpd36 and Cpd46, to eradicate MRSA and other staphylococci. Through whole-genome resequencing, we obtained several lines of evidence that these compounds might act by targeting the membrane protein translocase YidC2. Our data showed that ectopic expression of YidC2 in *S. aureus* decreased the bacterial susceptibility to Cpd36 and Cpd46, and that the YidC2-mediated tolerance to environmental stresses was suppressed by both compounds. Moreover, the membrane translocation of ATP synthase subunit c, a substrate of YidC2, was blocked by Cpd46, leading to a reduction in bacterial ATP production. Furthermore, we found that the thermal stability of bacterial YidC2 was enhanced, and introducing point mutations into the substrate-interacting cavity of YidC2 had a dramatic effect on Cpd36 binding via surface plasmon resonance assays. Finally, we demonstrated that these YidC2 inhibitors could effectively eradicate MRSA persisters and biofilms. Our findings highlight the potential of impeding YidC2-mediated translocation of membrane proteins as a new strategy for the treatment of bacterial infections.

## 1. Introduction

In bacterial cells, newly synthesized membrane proteins are inserted into membranes by the protein insertion machinery. Two conserved translocons have been identified in *Escherichia coli*: one is composed of Sec translocon and YidC, while the other one comprises only YidC [1,2]. YidC is involved in the insertion of several membrane proteins, including F_o_F_1_-ATPase subunits a and c, cytochrome *o* oxidase subunit a, and NADH-ubiquinone oxidoreductase subunit k [3,4,5]. As an important membrane protein translocase, YidC is essential for the growth of *E. coli* and is highly conserved among different bacteria [6]. In Gram-positive bacteria, YidC consists of two orthologues, namely, YidC1 and YidC2 [7]. Some species possess both YidC1 and YidC2, while others only have either YidC1 or YidC2. For example, *Streptococcus mutans* possesses both YidC1 and YidC2 [8], but *S. aureus* has YidC2 only [9]. Either YidC1 or YidC2 can restore the growth and F_o_F_1_ ATP synthase activity in the *E. coli yidC*-depletion strain, indicating that both of them are the functional complement of YidC [10]. Moreover, it has been shown that YidC1 and YidC2 cannot be concomitantly removed in *S. mutans*, suggesting that these two proteins are functional duplicates [8]. Although YidC1 and YidC2 are paralogues, elimination of *yidC2* in *S. mutans* results in a stress-sensitive phenotype, while deletion of *yidC1* has no obvious effect on growth or stress sensitivity [11]. In light of the essential role of YidC in bacterial cell growth, it has been proposed as a potential target for the development of new antibacterial agents [12,13].

We have previously identified several derivatives of the anti-inflammatory COX-2 inhibitor celecoxib, represented by Cpd9, Cpd36, and Cpd46 (structures, Figure S1) to exhibit high potency, at sub-µg/mL concentrations, in killing MRSA and other pathogenic staphylococci, while causing no appreciable toxicity to human cells. Moreover, a single intraperitoneal administration of Cpd46 at 30 mg/kg significantly improved the survival of MRSA-infected C57BL/6 mice [14]. In this study, we sought to gain insight into the mechanism of action of these anti-MRSA agents through mutational analysis in the chromosome of drug-resistant *S. aureus* isolates. The whole-genome resequencing data identified an essential membrane protein translocase, YidC2, as the potential target. Accordingly, the role of YidC2 in the antibacterial activity of Cpd36 and Cpd46, as well as the interaction of YidC2 with these agents, were the focus of this investigation.

## 2. Results

### 2.1. S. aureus Isolates with Resistance to Celecoxib-Derived Antibacterial Agents All Have Missense Mutations at yidC2

**Figure 1:**
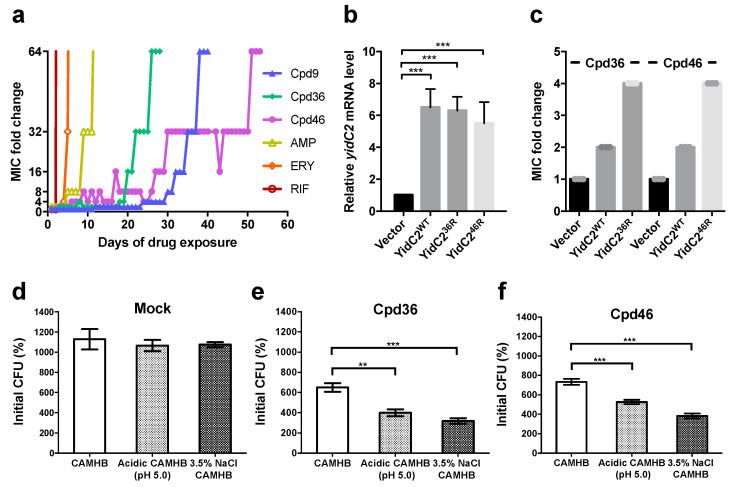
Celecoxib derivatives suppress *S. aureus* via targeting YidC2. (**a**) Drug-resistant *S. aureus* NCTC8325 isolates were selected by consecutively exposing bacteria to sub-lethal concentration of Cpd9, Cpd36, Cpd46, Ampicillin, Erythromycin and Rifampicin. (**b**) The levels of *yidC2* transcript of *S. aureus* NCTC8325 transformed with pRMC2, pRMC2-YidC2^WT^, pRMC2-YidC2^36R^ (P139L) and pRMC2-YidC2^46R^ (P73L) was analyzed by qRT-PCR after anhydrotetracycline (0.2 μg/mL) induction. (**c**) The susceptibility of transformed bacteria to Cpd36 and Cpd46 was determined by the MIC assay. Experiments were conducted thrice with three replicates per test. (**d**–**f**) To assess the stress-tolerance, *S. aureus* was treated with (**d**) mock, (**e**) Cpd36 or (**f**) Cpd46 in regular CAMHB for 1 h, washed with PBS and then cultured in regular CAMHB, acidic CAMHB (pH 5.0) or high-salt CAMHB (supplemented with 3.5% NaCl) for 12 h. The number of viable bacteria before and after stress-treatment was determined by the colony forming unit (CFU) assay. The CFU of bacteria culture before stress-treatment was considered as 100%. Data were presented as mean ± SD (n = 3). *p-*values were calculated by Student’s *t-*test. **, *p* < 0.01; ***, *p* < 0.001.

Previously, we identified several small-molecule agents, represented by Cpd9, Cpd36, and Cpd46, with potent anti-MRSA activities in vitro and in vivo [14]. To elucidate their mechanism of action, drug-resistant isolates were selected by continuously exposing *S. aureus* NCTC8325 to sub-lethal concentrations of Cpd9 and Cpd36 until the susceptibility of bacteria to individual compounds was decreased to 1/64 of that of the parental strain. As shown in Figure 1a, *S. aureus* rapidly developed resistance to conventional antibiotics, including ampicillin, erythromycin and rifampicin, within 10 days. Relatively, the resistance of *S. aureus* toward Cpd9 or Cpd36 was observed after 38 and 26 days of drug exposure, respectively, suggesting that these two compounds are less likely to induce resistance in *S. aureus.* Additionally, cross-resistance between these two compounds were observed (data not shown) due to structural similarities.

Next, mutation(s) that occurred in the chromosomes of Cpd9-resistant and Cpd36-resistant isolates was identified by whole-genome resequencing and comparing the chromosome sequence to that of the parental strain and the published whole-genome sequence of *S. aureus* NCTC8325 (NC_007795). The mutations identified were further validated using primer pairs (Table S2) to amplify the mutation-containing DNA regions on the chromosome followed by Sanger sequencing. There were seven point mutations identified in each chromosome of Cpd9-resistant and Cpd36-resistant *S. aureus* isolates, and these mutations occurred at both coding and non-coding regions of bacterial chromosomes (Table S3). It is noteworthy that in both isolates, missense mutations occurred in the *yidC2* gene, resulting in alterations in the corresponding coding amino acids located in the transmembrane domains of YidC2 protein (Figure S2 and Table S4).

To further validate the role of YidC2 in bacterial resistance to celecoxib-derived antibacterial agents, a Cpd46-resistant strain was isolated from an independent experiment after exposing bacterial cells to sublethal concentrations of Cpd46 for 53 days (Figure 1a). Similarly, the YidC2-coding sequence on the chromosome of Cpd46-resistant isolate was sequenced by the Sanger method, which also revealed a missense mutation in the *yidC2* gene (Figure S2 and Table S4). Together, these data suggested that YidC2 might be a potential target for these antibacterial agents. As Cpd36 and Cpd46 are more potent in killing *S. aureus* than Cpd9 [14], the role of YidC2 in the antibacterial activity of these two compounds is further investigated hereafter.

### 2.2. YidC2-Overexpressing Staphylococci are Less Susceptible to Cpd36 and Cpd46

To interrogate the role of YidC2 in mediating the antibacterial activity of these celecoxib derived agents, coding sequences of *yidC2* on the chromosomes of wild-type, Cpd36-resistant, and Cpd46-resistant *S. aureus* isolates were cloned into the downstream of a tetracycline regulated promoter on a shuttle vector, pRMC2 [15] and transformed into *S. aureus* NCTC8325. Bacterial cells harboring ectopically expressed wild-type YidC2 exhibited a 2-fold decrease in the susceptibility to Cpd36 and Cpd46 than those carrying empty plasmids (Figure 1b,c). The susceptibility to Cpd36 and Cpd46 was further reduced by the ectopic expression of mutated YidC2 from Cpd36-resistant isolate and Cpd46-resistant isolate, respectively, suggesting that YidC2 was involved in the antibacterial activity of these celecoxib derivatives (Figure 1c). Besides, the lower resistance observed in bacteria with ectopic expression of mutated YidC2 than that of resistant isolates selected by multiple-passages implicated that the YidC2 protein expressed from chromosomal *yidC2* gene might have better coordination with other bacterial proteins for membrane integration and function, making it more vulnerable to the celecoxib derivatives.

In addition to YidC2, some *Staphylococcus* species also have the YidC orthologue YidC1. By using the amino acid sequence of YidC2 as a reference, the copies of YidC1 and YidC2 orthologues were analyzed in different staphylococci. The results revealed that *S. aureus*, *S. epidermidis*, and *S. intermedius* had YidC2 only, while *S. haemolyticus*, *S. hominis*, *S. saprophyticus* and *S. lugdunensis* possessed both YidC1 and YidC2 (Table 1). It is noteworthy that *Staphylococcus* species harboring both YidC1 and YidC2 were less susceptible to Cpd36 and Cpd46 than those with YidC2 only (Table 1). As YidC1 and YidC2 are paralogues, we rationalized that Cpd36 and Cpd46 might suppress bacterial growth by suppressing the conserved function(s) of YidC1 and YidC2.

### 2.3. Cpd36 and Cpd46 Attenuate the Stress Tolerance of S. aureus

As a membrane translocase, YidC2 plays a pivotal role in regulating the insertion of a variety of membrane proteins, including those involved in bacterial tolerance to environmental stresses. For example, depletion of YidC2 was reported to decrease the tolerance of *S. mutans* to high-salt stress (osmotic stress) as well as acidic pH (acid stress) [11]. We also found that short exposure of bacterial cells to Cpd36 and Cpd46 resulted a significant reduction in bacterial growth in both acidic and high-salt mediums (Figure 1d–f), suggesting that these two compounds could attenuate the stress tolerance of bacterial cells by interfering with YidC2′s activity.

### 2.4. Cpd46 Blocks Membrane Insertion of F_o_F_1_ ATP Synthase Subunit C, Leading to Reduced ATP Production

**Figure 2:**
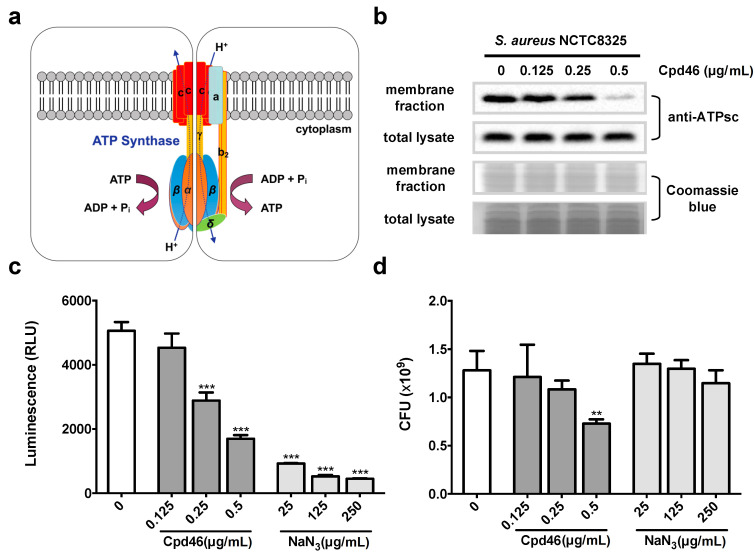
Cpd46 abrogates the membrane insertion of the F_o_F_1_ ATP synthase subunit c and reduced the intracellular ATP level of *S. aureus*. (**a**) Schematic diagram of ATP synthesis and hydrolysis by the ATP synthase complex driven by the movement of protons (dot arrows). (**b**) *S. aureus* NCTC8325 was exposed to various concentrations of Cpd46 and NaN3 in regular LB broth for 1 h. Levels of ATP synthase subunit c (ATPsc) in the total lysate and membrane fraction of bacteria were evaluated by immunoblotting. (**c**) The intracellular ATP level was measured by the luciferase-based ATPlite one-step assay. (**d**) The viability of bacteria was evaluated by the CFU assay. Data were presented as mean ± SD (n = 3). The differences between Cpd46-treated, NaN3-treated and untreated samples was calculated using Student’s *t-*test. **, *p* < 0.01; ***, *p* < 0.001.

The F_o_F_1_ ATP synthase consists of several membrane proteins, and is responsible for the production of ATP, an important energy carrier molecule in bacteria [16]. Among individual subunits of the ATP synthase, the subunits a and c are inserted into bacterial cytoplasmic membrane by YidC translocon (Figure 2a) [3]. As shown in Figure 2b, the level of membrane-bound ATP synthase subunit c (ATPsc) decreased in a concentration-dependent manner in Cpd46-treated cells, indicating the suppressive effect of Cpd46 on the insertion of this YidC2-dependent membrane protein.

Bacterial F_o_F_1_ ATP synthase produces ATP from ADP and inorganic phosphate by using the ionic gradient as a driving force [16]. Thus, the ability of ATP synthase to produce ATP requires the assembly of its complex in cytoplasmic membranes (Figure 2a). To verify the impact of Cpd46-mediated suppression of ATPsc insertion on ATP production, we measured the intracellular ATP levels in vehicle- vs. Cpd46-treated bacterial cells together with the known ATP synthesis inhibitor sodium azide (NaN_3_) as a positive control [17]. Consistent with our premise, Cpd46 at concentrations of 0.25 μg/mL and above could significantly suppress intracellular ATP production (*p* < 0.001; Figure 2c). The above data provide evidence that the disruption of YidC2-mediated ATPsc membrane insertion represents a major mechanism by which these celecoxib derivatives mediated their anti-MRSA activity. As YidC2′s targets consist of multiple membrane proteins, it is most likely that the anti-MRSA action of these small-molecules would be attributable to the concerted effects on multiple proteins. For example, ATP depletion alone, as exemplified by the effect of NaN_3_ on cell viability (Figure 2d), was insufficient to account for the anti-MRSA activity of Cpd46.

### 2.5. Cpd36 and Cpd46 Enhance the Thermal Stability of YidC2

**Figure 3:**
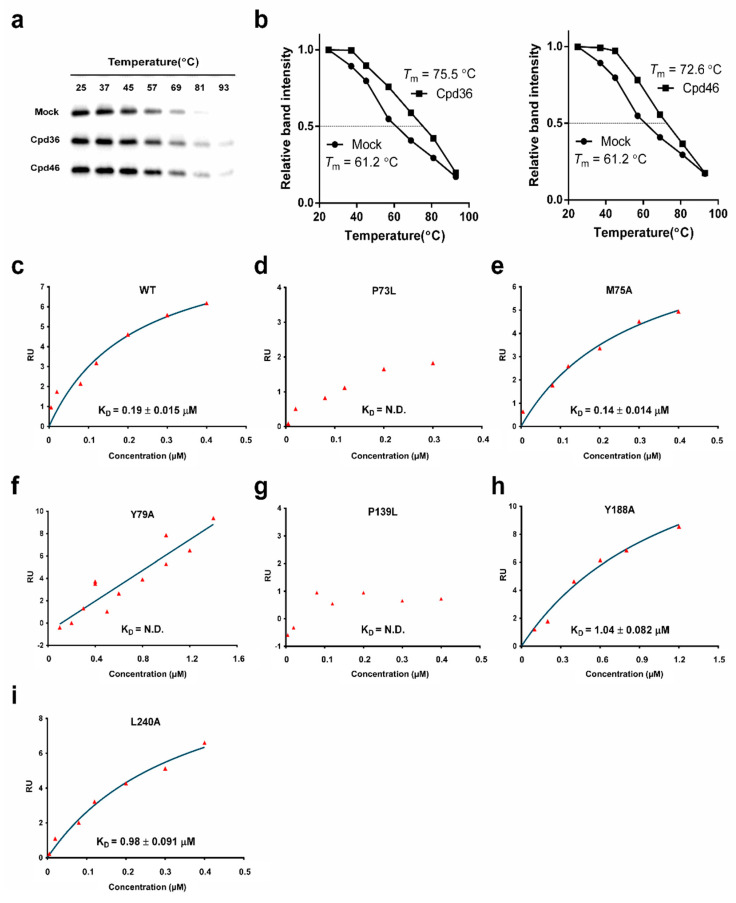
The interaction of YidC2 and Cpd36 is abolished by the mutations at substrate-contacting groove. The thermal stability of *S. aureus* YidC2 was measured by treating bacterial lysate with Cpd36 (10 μg/mL) and Cpd46 (10 μg/mL), respectively, followed by incubation at escalating temperature ranging from 25 °C to 93 °C. (**a**) The level of YidC2 in individual samples was assessed by immunoblotting. (**b**) The relative intensities of Cpd36-treated or Cpd46-treated signals were quantitated using ImageJ software and normalized to the signal of 25 °C, respectively. (**c**–**i**) Fitting of steady-state responses from the interaction of recombinant wild-type and mutated of *S. aureus* YidC2 and Cpd36 were measured by surface plasmon resonance to determine the K_D_ value. The steady-state signal reached at the end of the analyte injection (120 s at 30 µL/min) was plotted against the analyte concentration and the resulting curve fitted with 1:1 binding model. Abbreviation: N.D., not determined.

The above findings raised the question of whether Cpd36 and Cpd46 interacted with YidC2 protein through direct binding. To address this issue, we conducted the thermal shift assay, in which the effect of ligand binding on the thermal stability of a target protein was analyzed [18]. As shown, the melting temperatures (*T*_m_) of YidC2 treated with vehicle, Cpd36 or Cpd46 are 61.2, 75.5 and 72.6 °C, respectively (Figure 3a,b). With the T*m* values increasing by more than 10 °C, these results raised the possibility that Cpd36 and Cpd46 could increase YidC2 protein stability through direct interactions.

### 2.6. Identification of the Drug-Interacting Motif on YidC2 via Homology Modeling and Mutational Analyses

**Figure 4:**
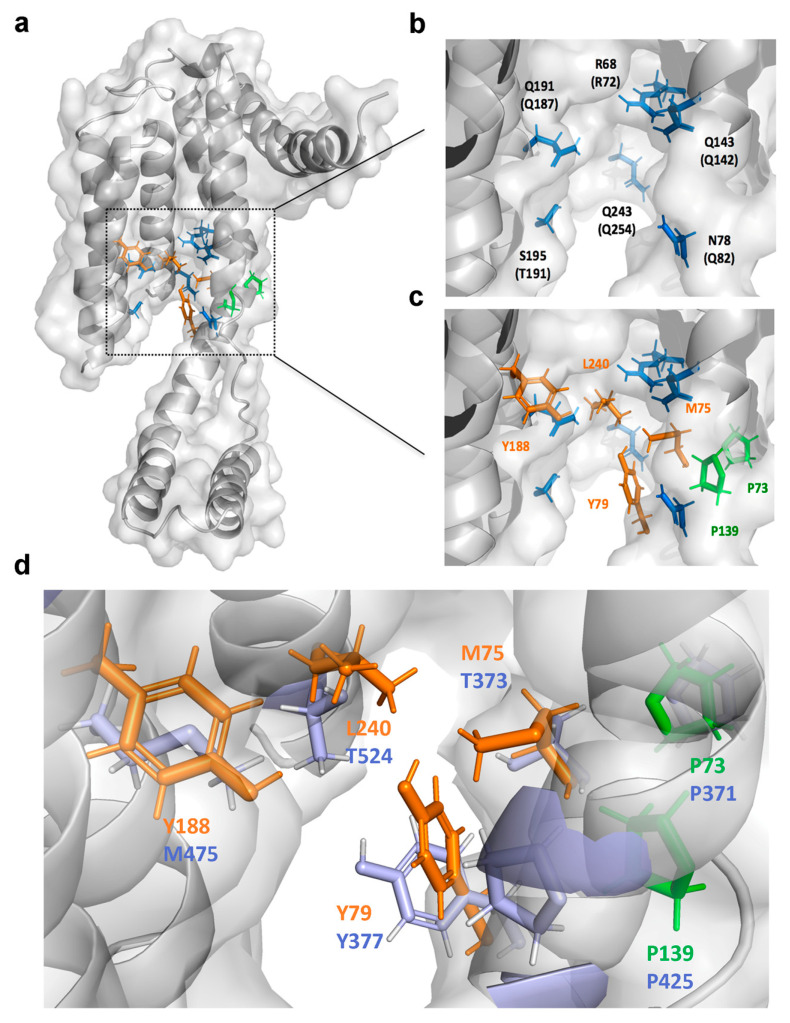
Substrate-contacting cavity of *S. aureus* YidC2. (**a**) The homology model of *S. aureus* YidC2 was built based on X-ray structures of *B. halodurans* YidC2 (PDB code: 3WO6 and 3WO7). (**b**) The hydrophilic groove of YidC is found to be conserved and the corresponding resides in *B. halodurans* YidCs are indicated in parentheses. (**c**) Mutations of YidC2 identified from drug-resistant *S. aureus* isolates are shown in green. The hydrophobic residues in the groove are shown in orange. (**d**) Comparison of Cpd36-contacting residues of YidC2 in *S. aureus* and *E. coli* shown in orange and purple, respectively.

In order to identify the drug-binding motif on YidC2, we first conducted homology modeling to gain insight into the mode of binding of Cpd36 into YidC2. To date, the crystal structures of YidC from *Bacillus halodurans* (BhYidC2) and *E. coli* (EcYidC) have been reported at 2.4 and 3.2 Å resolution, respectively [19,20]. Based on the currently available high-resolution structures of YidC2, the architectures of the core five TM helixes are essentially identical. Due to the difficulty in crystallizing YidC2 from *S. aureus* (SaYidC2), we used the crystal structures of YidC from *Bacillus halodurans* (PDB code: 3WO7) as a template and generated the 3D model structure of SaYidC2 by Swiss Model [21] as these two YidC2 proteins share 40.4% identity in the architecture of the core TM region (Figure 4a,b). Several substrate-contacting residues of EcYidC and BhYidC2 have been identified by cross-linking analysis using Sec-independent substrates, including MifM, Pf3 coat protein, and subunit c of the ATP synthase [19,22,23]. These residues are located primarily on the exterior surface of the TM region and the interior surface of the hydrophilic groove, indicating that the substrate-contacting residues of YidC proteins are clustered in the area close to the groove. These observations underpinned the importance of both the hydrophobic and hydrophilic interactions between substrates and YidC in mediating the Sec-independent protein insertion (Figure 4c) [19]. Interestingly, the two proline residues identified at YidC2 of the Cpd36-resistant and Cpd46-resistant isolates are conserved and quite close to the substrate-contacting site: Pro73 introduces a kink at TM1 helix and Pro139 is located at a flexible loop connecting the CH2 and TM2. To explore the role of these two conserved proline residues and neighboring hydrophobic residues, we conducted a mutational analysis by generating a series of SaYidC2 variants with point mutations, including P73L, M75A, Y79A, P139L, Y188A, and L240A. SPR analysis was revealed that the binding affinity (K_D_) of wild-type YidC2 and Cpd36 was 0.19 ± 0.015 µM and point mutations at most of these hydrophobic residues substantially weakened (Y188A and L240A) or abolished (P73L, P139L, and Y79A) this ligand binding (Figure 3c–i and Figure S3), while the binding affinity of M75A mutant remained relatively unchanged, as did that of wild-type. Together, these data suggest the importance of these hydrophobic residues in the substrate-contacting groove in mediating Cpd36–SaYidC2 interactions.

### 2.7. Cpd46 Effectively Eradicates MRSA Persisters and Biofilms

**Figure 5:**
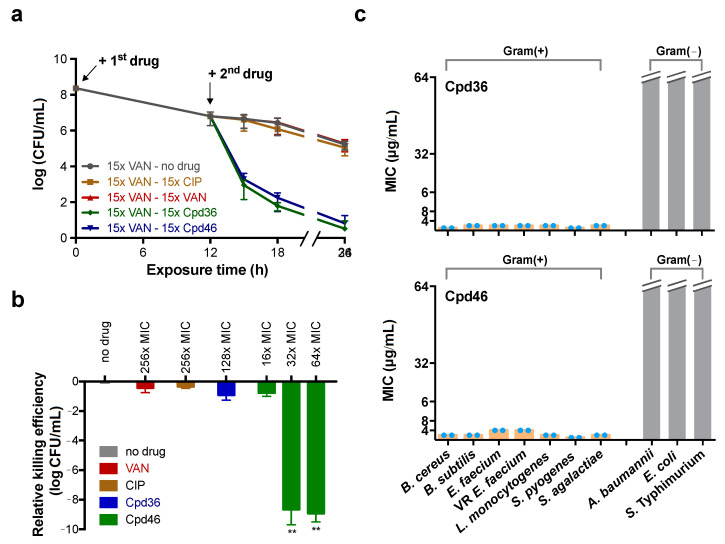
Cpd46 is effective against Gram-positive bacteria, persisters and biofilms. (**a**) The killing kinetics of ciprofloxacin, vancomycin, Cpd36, and Cpd46 on MRSA ATCC33592 persisters induced by 15 μg/mL (15× MIC) of vancomycin. Data shown are the mean and standard deviation of three biological replicates. (**b**) The eradicating activity of Cpd36, Cpd46 and antibiotics against MRSA ATCC33592 biofilms was assessed by the biofilm eradication assay. Each viable cell number is expressed as log CFU/mL, and Y-axis values reflect the reduced cell numbers compared to that of the drug-free one. Data are expressed as mean ± SD, *n* = 3. The differences between drug-treated and untreated samples was calculated using Dunn’s multiple comparison test. **, *p* < 0.01. (**c**) The susceptibility of a collection of Gram-positive and Gram-negative bacteria to Cpd36 and Cpd46 were assessed using the MIC assay. Experiments were conducted twice (blue dots) with three replicates per treatment set.

The phenomenon of persistence is where a small population of bacteria enter into a non-growing state and develop tolerance to antibiotic treatment, leading to recurrent bacterial infections [24]. Given that many YidC-dependent membrane proteins play a crucial role in maintaining the integrity of cell morphology and/or bioenergetics, we hypothesized that YidC2 might be essential to the survival of *S. aureus* cells with lower metabolic turnover. As shown in Figure 5a, the vancomycin-induced persisters were resistant to the subsequent challenge of vancomycin or ciprofloxacin. In contrast, they remained highly susceptible to Cpd36 and Cpd46, as reflected by a greater than 5-log reduction in the number of viable bacterial cells after 24 h of drug exposure, suggesting that YidC2 represents a promising anti-persister target.

Equally importantly, persisters have also been reported to exist in the biofilms, which are composed of attached microorganisms enclosed in an extracellular polymeric substance matrix [25] and can protect bacteria from antibiotic-mediated killing, as shown by the ineffectiveness of ciprofloxacin and vancomycin to clear bacteria inside biofilm even at 256× MIC (Figure 5b). Although both Cpd36 and Cpd46 are capable of killing persisters, only Cpd46 had a detectable activity in eradicating bacteria in the biofilms, suggesting the possibility that Cpd46 might be able to penetrate the biofilm barrier to kill persister cells (Figure 5b).

### 2.8. Cpd36 and Cpd46 are Active against Other Gram-Positive Bacteria

As the functions of the proteins in the YidC family are highly conserved among different bacterial genera [6,10], we further interrogated the effectiveness of Cpd36 and Cpd46 in other bacteria. The homology analysis revealed that the identity and similarity of YidC2 from different genera of Gram-positive bacteria were in the ranges 28–36% and 47–58%, respectively. In contrast, *S. aureus* YidC2 shares a low degree of homology with Gram-negative bacteria YidC, i.e., less than 13% and 23% in identity and similarity, respectively (Table S5). Subsequent growth inhibition assays also showed that Cpd36 and Cpd46 were effective against Gram-positive bacteria but exhibited no detectable inhibitory activity toward Gram-negative bacteria (Figure 5c). Moreover, no suppressive effect of Cpd36 and Cpd46 on the growth of *E. coli* strains defective in the efflux pump or outer membrane, was observed (Table S6). Together with our modeling analysis (Figure 4d), this suggests differences in the mode of interaction of these two compounds with YidC2 in *S. aureus* versus that of *E. coli*. Most notable were the four semi-contiguous Cpd36-binding residues identified in YidC2 of *S. aureus* (M75, Y79, Y188, and L240), whereas those of *E.* *coli* YidC are aromatic (Y377), aliphatic (M475) and hydrophilic (T373 and T524) residues (Figure 4d). These differences might shed light on the design of effective inhibitors of *E. coli* YidC, which is currently underway.

## 3. Discussion

The emergence of multiple antibiotic-resistant bacteria underscores the importance of identifying new druggable targets to facilitate antibiotic discovery. Previously, we reported a series of novel celecoxib derivatives with potent antibacterial activities against MRSA and other *Staphylococcus* pathogens [14]. In this study, we embarked on elucidating the mechanism by which these small-molecule agents, represented by Cpd36 and Cpd46, mediated their anti-MRSA effect using a multi-disciplinary approach. We obtained several lines of evidence that Cpd36 and Cpd46 suppressed bacterial cell growth by blocking YidC2-mediated ATPsc membrane insertion. Furthermore, homology modeling analysis, in conjunction with mutational analysis, suggests that Cpd36 and Cpd46 might interact with YidC2 through the hydrophobic region in the substrate-interacting groove. From a translational perspective, the impetus of this study is multifold. For example, this study obtained the first evidence that YidC2 is a druggable target for the development of new anti-MRSA agents. Second, Cpd36 and Cpd46 are amenable to chemical modifications, thereby providing a good starting point for designing more potent YidC2 inhibitors, via a structure-based strategy, for MRSA treatment.

The TM1 helix of SaYidC2 is kinked at the conserved Pro73 and Pro94 (Pro77 and P94 in BhYidC2; Pro371 and Pro388 in EcYidC). Helix kinks are important as they are flexible and/or carry out crucial functional roles and loss of proline from a kinked helix often also results in the loss of a kink or reduction in its kink angle. P73L-resistant mutation is expected to alter the structure of the transmembrane domain and the vicinity of the substrate-contacting site. Pro139 is also conserved at a flexible loop connecting the CH2 and TM2 in BhYidC2 (Pro138) and EcYidC (Pro425). Proline is considered to be an alpha-helix breaker and the P139L may introduce backbone hydrogen-bonding and result in perturbation in structure. Indeed, our steady-state analysis of SPR data has shown that the replacement of Pro73 or Pro139 with Leu abolished the interaction of Cpd36 and SaYidC2. Furthermore, Cpd36- or Cpd46-binding to YidC2 of *S. aureus* led to increases in T*m* values by more than 10 °C, suggesting that Cpd36 or Cpd46 could reduce the structural flexibility of YidC2 protein by occupying the substrate-binding cavity. Together, these structural data provide useful information for the lead optimization of Cpd36 and Cpd46 to develop potent YidC2 inhibitors, which is currently underway in this laboratory.

## 4. Materials and Methods

### 4.1. Bacterial Strains

Bacteria used in this work were listed in Supplementary Materials Table S1.

### 4.2. Reagents and Antibodies

The details of the synthesis of celecoxib derivatives have been reported in Chiu et al., 2012 [14]. The purities of all tested compounds were determined to be 95% by using proton NMR (1H-NMR) spectra with DPX400 (400 MHz, Bruker, Billerica, MA, USA) instruments. Rabbit anti-YidC2 and anti-ATPsc polyclonal antibodies are customized products from GeneTex (Hsinchu, Taiwan).

### 4.3. Antibacterial Assay

The minimal inhibitory concentration (MIC) of each compound was determined as described previously [26]. Briefly, bacteria grown on Luria Bertani (LB) plates or blood agar plates (Dr. Plate Biotech Company, Taipei, Taiwan) were inoculated in cation-adjusted Mueller Hinton broth (CAMHB) or brain heart infusion broth to a final concentration of 5 × 10^5^ CFU/mL. Bacteria were then exposed to the test compounds and chloramphenicol at escalating concentrations, ranging from 0.25 to 16 μg/mL, in triplicate in 96-well plates at 37 °C for 24 h. The MIC of the individual compound was defined as the lowest concentration at which no visible growth of bacteria was observed.

### 4.4. Isolation of Drug-Resistant S. aureus

*S. aureus* NCTC8325 was first exposed to escalating doses of drugs followed the MIC assay protocol described above. After 24 h, bacteria in the well with drug concentration at 0.5× MIC were 1:500 diluted in fresh CAMHB followed by exposure to escalating doses of the same test compound for another 24 h. The experiment was repeated every 24 h until the susceptibility of bacteria to test compound is decreased to 1/64 of that at the initial point, as reflected by a 64-fold increase in the MIC value. To validate the stability of resistance, bacteria were sub-cultured on a drug-free CAMH agar plate and incubated at 37 °C for 24 h. After successive sub-cultures on a drug-free CAMH agar plate seven times, the susceptibility of bacteria to test drugs was examined using MIC assay.

### 4.5. Genomic Mutations Identification

Mutations occurred in the chromosomes of drug-resistant *S. aureus* isolates were identified with whole-genome resequencing service provided by NGS and Microarray Core Lab, NTU Center of Genomic Medicine (Taipei, Taiwan). Briefly, bacterial genomic DNA was sheared, end-repaired, and ligated with sequencing adaptors. The adaptor-ligated DNA fragments were amplified to produce the DNA library and the quantity and quality of the library were determined by Qubit 2.0 (Thermo Fisher Scientific, Waltham, MA, USA) and Bioanalyzer (Agilent Technologies, Santa Clara, CA, USA), respectively. The DNA library was then subjected to standard sample preparation procedures as recommended by the manufacturer and sequenced using the Sequencing by Oligo Ligation and Detection 5500 system (Applied Biosystems, Foster City, CA, USA). Reads were aligned against the *S. aureus* NCTC8325 genome sequence (NC_007795) published on the National Center for Biotechnology Information (NCBI) using the LifeScope software (v1.0, Applied Biosystems). Only those mapped to a unique position in the reference genome were used for single-nucleotide variation (SNV) and small insertion/deletion (InDel) calling. Mutations were identified and then annotated by ANNOVAR program [27].

### 4.6. Identification of YidC2 Orthologue

The search of YidC2 homologues in staphylococci was performed following the procedure reported by Zhang et al. [28]. Briefly, protein sequence of the YidC2 of *S. aureus* NCTC8325 (YP_500806.1) was used as a query to search against protein annotations of selected *Staphylococcus* species in the genome databases in NCBI, with an E value of 0.07 as a cutoff to obtain putative homologs. Only those with the best hits in BLASTP against the NCBI non-redundant database to known YidC/YidC1/YidC2 were considered as orthologues of *S. aureus* YidC2.

### 4.7. Thermal Shift Assay

Bacteria were lysed by sonication with the 550 Sonic Dismembrator (Thermo Fisher Scientific) for a total of 10 min (10 s ON/ 10-s OFF pulses) on ice and treated with Cpd36, Cpd46, or DMSO. After 60 min incubation at 37 °C, the lysates were centrifuged at 10,000× *g* for 20 min at 4 °C, and the soluble fraction was collected and incubated at temperatures ranging from 25 °C to 93 °C for 30 min, followed by cooling at 25 °C for 3 min. Heated lysates were centrifuged at 16,000× *g* at 4 °C for 20 min and the level of YidC2 in the supernatants was detected by immunoblotting.

### 4.8. Isolation of Bacterial Membrane Proteins

After exposure to test compound for 60 min, bacteria were lysed in 20 mM Tris (pH 8.0) supplemented with lysostaphin (1 µg/mL) at 37 °C for 2 h and then sonicated with 550 Sonic Dismembrator (Thermo Fisher Scientific) for a total of 2 min (10 s ON/10 s OFF pulses) on ice. The cell debris was removed by centrifugation at 10,000× *g* for 10 min at 4 °C. The membrane fraction was collected by centrifugation at 120,000× *g* for 45 min at 4 °C (Optima™ L-100K Ultracentrifuge, Beckman Coulter, Brea, CA, USA), and solubilized in 20 mM Tris buffer, 300 mM NaCl, 0.1–1% n-Dodecyl-β-D-maltoside (DDM), 0.01% cholesteryl hemisuccinate (CHS), pH 8.0.

### 4.9. Intracellular ATP Measurement

After treating with test compounds for 60 min, bacteria were disrupted by vigorous vortex with 100–150 µm diameter glass beads followed by centrifugation at 12,000× *g* for 15 min at 4 °C to remove the debris. The intracellular ATP level was determined using the ATPlite one-step assay kit (Perkin Elmer, Waltham, MA, USA) and measured by the Spectromax M5 microplate reader (Molecular Devices, San Jose, CA, USA).

### 4.10. Surface Plasmon Resonance (SPR) Analysis

*S. aureus* YidC2 (YidC) was cloned into a pET-modified vector [19] and mutants (P73L, M75A, P79L, P139L, Y188A, L240A) were generated by PCR-based site-directed mutagenesis approach with primers listed in Supplementary Materials Table S2. The recombinant 6-histidine tagged YidC2 was purified with Ni-NTA Superflow (Qiagen, Hilden, North Rhine-Westphalia, Germany) and eluted with 300 mM NaCl, 20 mM Tris-HCl (pH 8.0), 0.1% DDM and 0.01% CHS, supplemented with imidazole at concentrations up to 300 mM at 4 °C. The protein was concentrated and further purified on a Superdex 200 10/300 GL column (GE Healthcare, Chicago, IL, USA) in 300 mM NaCl, 20 mM Tris-HCl, pH 8.0, 0.1% DDM and 0.01% CHS at 4 °C. Binding experiments were performed on a Biacore T200 instrument (GE Healthcare, Piscataway, NJ, USA). In order to increase capture stability on sensor chip NTA, we performed an amine coupling of the captured YIdC2 variants. To evaluate the binding between SaYidC2 protein variants and celecoxib derivatives, the recombinant proteins were immobilized on an NTA sensor chip. The celecoxib derivatives were diluted in SPR buffer (2.5% DMSO, 25 mM NaPi pH 7.4, 300 mM NaCl, 0.1% DDM, 0.01% CHS, 0.05% Tween20) to different concentrations and injected at a flow rate of 30 μL/min. K_D_ values were determined using the steady-state fitting with a one-site binding model.

### 4.11. Persisters Killing Assay

Overnight grown bacteria were 1:50 diluted into fresh LB broth and cultured at 37 °C to early stationary phase of growth (OD_600_ = 1.0). Bacteria were then treated with vancomycin (Goldbio, St. Louis, MO, USA) at 15× MIC at 37 °C for 12 h and collected by centrifugation at 4000× *g* for 10 min at 4 °C. After washing with ice-cold PBS, bacteria were suspended in PBS followed by the addition of Cpd36, Cpd46, vancomycin, or ciprofloxacin (Sigma-Aldrich, St. Louis, MO, USA) at 15× MIC. Controls received DMSO vehicle at a concentration equal to that in drug-treated bacteria. At the designed times, the number of viable bacteria was assessed by the CFU assay and expressed as CFU per milliliter.

### 4.12. Biofilm Eradication Assay

The eradicating activity of antibacterial agents against bacteria in the biofilms was assessed as described previously [29]. After drug treatment, the biofilms on the pegs were dislodged into fresh CAMHB in a 96-well microplate (Greiner, Kremsmünster, Austria) and incubated for 24 h at 37 °C. The number of viable bacteria in each well was assessed by the CFU assay and expressed as CFU per milliliter.

### 4.13. Quantification and Statistical Analysis

At least two independent experiments were performed with three biological replicates. Data are expressed as means ± the standard deviation. Differences between group means were calculated using a two-tailed Student’s *t*-test for independent samples and were considered significant at *p* < 0.05. All statistical analysis was performed using Graphpad Prism software (v7.0, San Diego, CA, USA).

## Figures and Tables

**Table 1:** Susceptibility of *Staphylococcus* species to Cpd36 and Cpd46 versus the presence of *yidC1* and *yidC2* at bacterial chromosome.

	MIC of Cpd36 (μg/mL)	MIC of Cpd46 (μg/mL)	*yidC1*	*yidC2*
*S. aureus* (ATCC12598)	1	0.5	−	+
*S. epidermidis* (ATCC12228)	1	0.5	−	+
*S. haemolyticus* (ATCC29970)	2	2	+	+
*S. hominis* (ATCC27844)	2	2	+	+
*S. intermedius* (ATCC29663)	1	0.5	− ^a^	+
*S. saprophyticus* (ATCC15305)	2	2	+	+
*S. lugdunensis* (NTUH isolate)	2	2	+	+

Data was obtained by using BLASTP to identify *S. aureus* NCTC8325 *yidC2* homologues starting in July 2019 and an e-value of 0.07 as the cutoff. ^a^.191 a.a., e-value: 8 × 10^−68^.

## Supplementary Materials

Supplementary materials can be found at https://www.mdpi.com/1422-0067/21/23/9312/s1.

## Abbreviations

MRSA	methicillin-resistant *S. aureus*
ATPsc	ATP synthase subunit c
*T*_m_	melting temperature
MIC	minimal inhibitory concentration

## References

[1] Samuelson J.C., Chen M., Jiang F., Moller I., Wiedmann M., Kuhn A., Phillips G.J., Dalbey R.E. (2000). YidC mediates membrane protein insertion in bacteria. Nature.

[2] Kuhn A., Koch H.G., Dalbey R.E. (2017). Targeting and Insertion of Membrane Proteins. EcoSal Plus.

[3] Yi L., Jiang F., Chen M., Cain B., Bolhuis A., Dalbey R.E. (2003). YidC is strictly required for membrane insertion of subunits a and c of the F(1)F(0)ATP synthase and SecE of the SecYEG translocase. Biochemistry.

[4] Du Plessis D.J., Nouwen N., Driessen A.J. (2006). Subunit a of cytochrome o oxidase requires both YidC and SecYEG for membrane insertion. J. Biol. Chem..

[5] Price C.E., Driessen A.J. (2010). Conserved negative charges in the transmembrane segments of subunit K of the NADH:ubiquinone oxidoreductase determine its dependence on YidC for membrane insertion. J. Biol. Chem..

[6] Van Bloois E., Nagamori S., Koningstein G., Ullers R.S., Preuss M., Oudega B., Harms N., Kaback H.R., Herrmann J.M., Luirink J. (2005). The Sec-independent function of Escherichia coli YidC is evolutionary-conserved and essential. J. Biol. Chem..

[7] Funes S., Kauff F., van der Sluis E.O., Ott M., Herrmann J.M. (2011). Evolution of YidC/Oxa1/Alb3 insertases: Three independent gene duplications followed by functional specialization in bacteria, mitochondria and chloroplasts. Biol. Chem..

[8] Palmer S.R., Crowley P.J., Oli M.W., Ruelf M.A., Michalek S.M., Brady L.J. (2012). YidC1 and YidC2 are functionally distinct proteins involved in protein secretion, biofilm formation and cariogenicity of Streptococcus mutans. Microbiology.

[9] Chiba S., Lamsa A., Pogliano K. (2009). A ribosome-nascent chain sensor of membrane protein biogenesis in Bacillus subtilis. EMBO J..

[10] Dong Y., Palmer S.R., Hasona A., Nagamori S., Kaback H.R., Dalbey R.E., Brady L.J. (2008). Functional overlap but lack of complete cross-complementation of Streptococcus mutans and Escherichia coli YidC orthologs. J. Bacteriol..

[11] Hasona A., Crowley P.J., Levesque C.M., Mair R.W., Cvitkovitch D.G., Bleiweis A.S., Brady L.J. (2005). Streptococcal viability and diminished stress tolerance in mutants lacking the signal recognition particle pathway or YidC2. Proc. Natl. Acad. Sci. USA.

[12] Patil S.D., Sharma R., Srivastava S., Navani N.K., Pathania R. (2013). Downregulation of yidC in Escherichia coli by antisense RNA expression results in sensitization to antibacterial essential oils eugenol and carvacrol. PLoS ONE.

[13] Hofbauer B., Vomacka J., Stahl M., Korotkov V.S., Jennings M.C., Wuest W.M., Sieber S.A. (2018). Dual Inhibitor of Staphylococcus aureus Virulence and Biofilm Attenuates Expression of Major Toxins and Adhesins. Biochemistry.

[14] Chiu H.C., Lee S.L., Kapuriya N., Wang D., Chen Y.R., Yu S.L., Kulp S.K., Teng L.J., Chen C.S. (2012). Development of novel antibacterial agents against methicillin-resistant Staphylococcus aureus. Bioorg. Med. Chem..

[15] Corrigan R.M., Foster T.J. (2009). An improved tetracycline-inducible expression vector for Staphylococcus aureus. Plasmid.

[16] Guo H., Suzuki T., Rubinstein J.L. (2019). Structure of a bacterial ATP synthase. Elife.

[17] Bald D., Amano T., Muneyuki E., Pitard B., Rigaud J.L., Kruip J., Hisabori T., Yoshida M., Shibata M. (1998). ATP synthesis by F0F1-ATP synthase independent of noncatalytic nucleotide binding sites and insensitive to azide inhibition. J. Biol. Chem..

[18] Jafari R., Almqvist H., Axelsson H., Ignatushchenko M., Lundback T., Nordlund P., Martinez Molina D. (2014). The cellular thermal shift assay for evaluating drug target interactions in cells. Nat. Protoc..

[19] Kumazaki K., Chiba S., Takemoto M., Furukawa A., Nishiyama K., Sugano Y., Mori T., Dohmae N., Hirata K., Nakada-Nakura Y. (2014). Structural basis of Sec-independent membrane protein insertion by YidC. Nature.

[20] Kumazaki K., Kishimoto T., Furukawa A., Mori H., Tanaka Y., Dohmae N., Ishitani R., Tsukazaki T., Nureki O. (2014). Crystal structure of Escherichia coli YidC, a membrane protein chaperone and insertase. Sci. Rep..

[21] Waterhouse A., Bertoni M., Bienert S., Studer G., Tauriello G., Gumienny R., Heer F.T., de Beer T.A.P., Rempfer C., Bordoli L. (2018). SWISS-MODEL: Homology modelling of protein structures and complexes. Nucleic Acids Res..

[22] Klenner C., Kuhn A. (2012). Dynamic disulfide scanning of the membrane-inserting Pf3 coat protein reveals multiple YidC substrate contacts. J. Biol. Chem..

[23] Yu Z., Koningstein G., Pop A., Luirink J. (2008). The conserved third transmembrane segment of YidC contacts nascent Escherichia coli inner membrane proteins. J. Biol. Chem..

[24] Lewis K. (2010). Persister cells. Annu. Rev. Microbiol..

[25] Wood T.K., Knabel S.J., Kwan B.W. (2013). Bacterial persister cell formation and dormancy. Appl. Environ. Microbiol..

[26] Chang H.C., Huang Y.T., Chen C.S., Chen Y.W., Huang Y.T., Su J.C., Teng L.J., Shiau C.W., Chiu H.C. (2016). In vitro and in vivo activity of a novel sorafenib derivative SC5005 against MRSA. J. Antimicrob. Chemother..

[27] Wang K., Li M., Hakonarson H. (2010). ANNOVAR: Functional annotation of genetic variants from high-throughput sequencing data. Nucleic Acids Res..

[28] Zhang Y.J., Tian H.F., Wen J.F. (2009). The evolution of YidC/Oxa/Alb3 family in the three domains of life: A phylogenomic analysis. BMC Evol. Biol..

[29] Su J.C., Huang Y.T., Chen C.S., Chiu H.C., Shiau C.W. (2017). Design and Synthesis of Malonamide Derivatives as Antibiotics against Methicillin-Resistant Staphylococcus aureus. Molecules.

