# Supplementary material for: A Celecoxib Derivative Eradicates Antibiotic-Resistant Staphylococcus aureus and Biofilms by Targeting YidC2 Translocase
Source: Int J Mol Sci. 2020 Dec 7;21(23):9312. doi: 10.3390/ijms21239312 (PMC7730571; doi:10.3390/ijms21239312)
Supplement: Supplementary file 1 [file ijms-21-09312-s001.pdf]

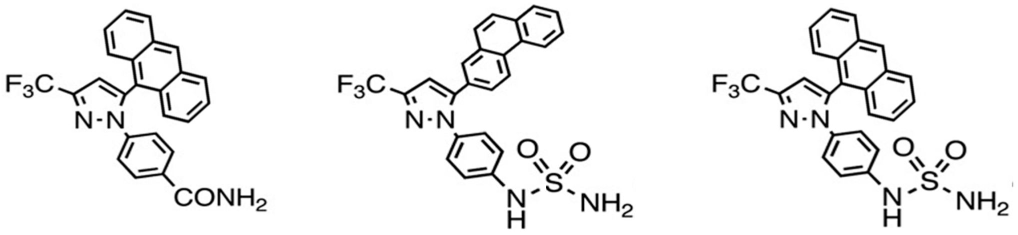

Cpd9 Cpd36 Cpd46

Figure S1. The structures of Cpd9, Cpd36 and Cpd46.

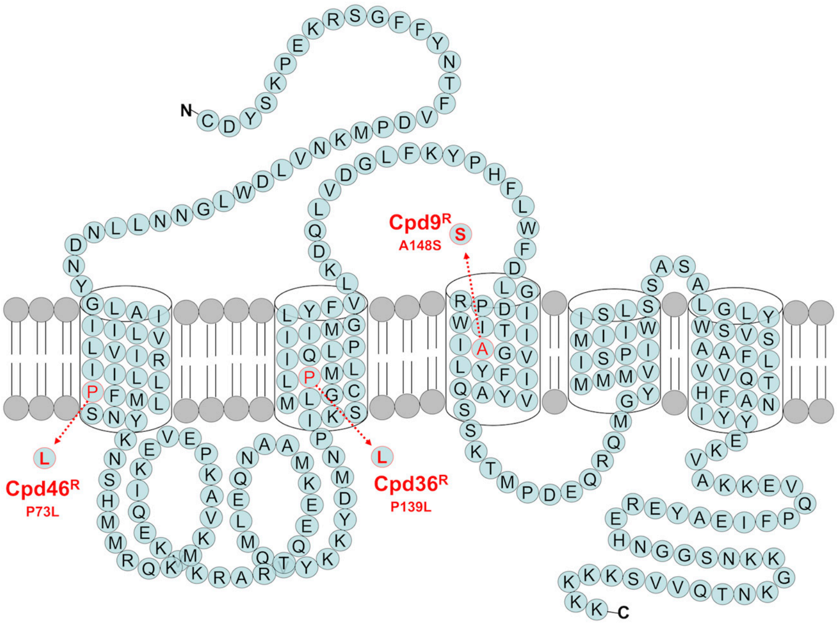

Figure S2. Missense mutations identified in YidC2 of drug-resistant *S. aureus* isolates.

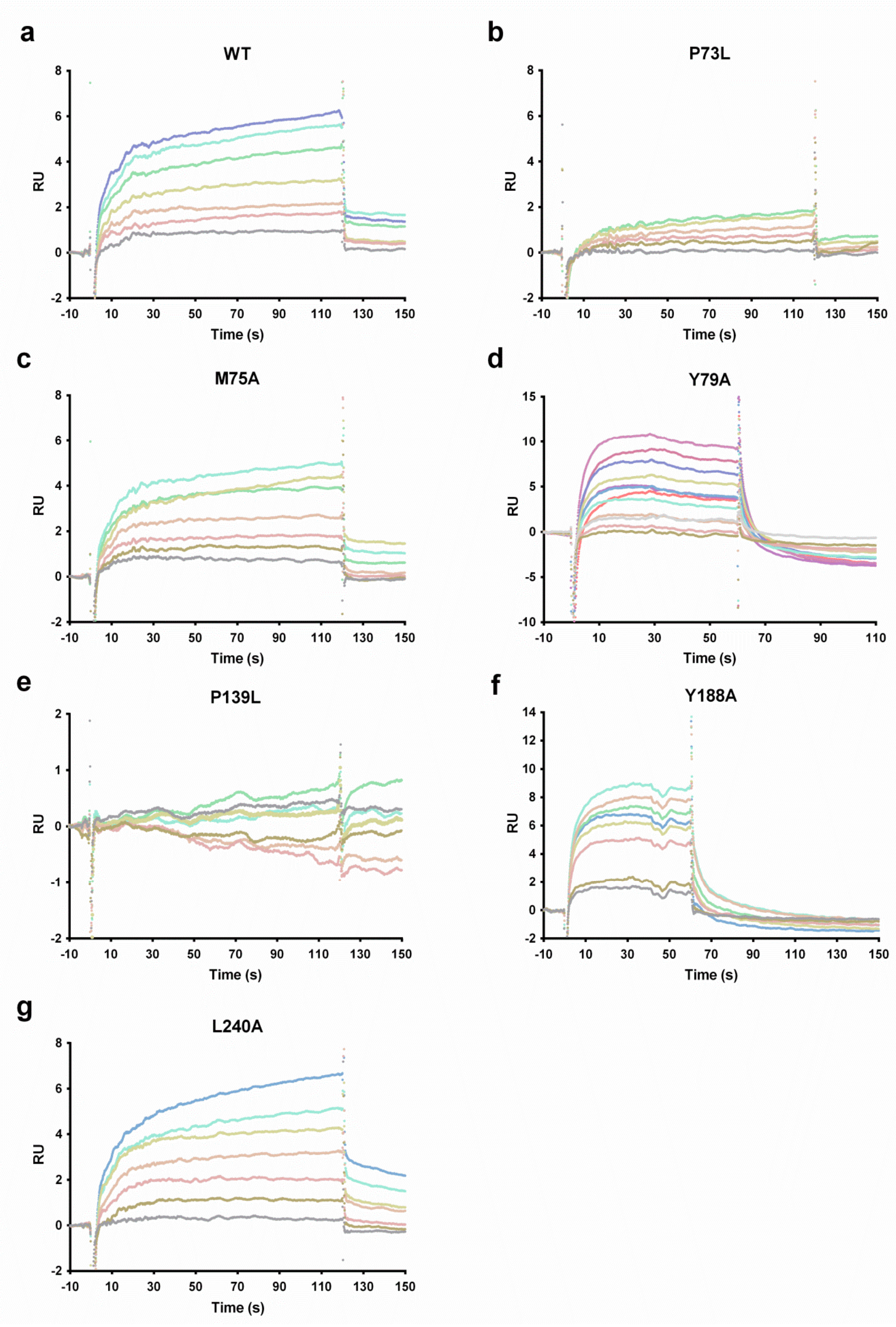

Figure S3. Steady-state analysis of SPR data: affinity values for SaYidC2 variants with Cpd36. Fitting of steady-state responses from SaYidC2-Cpd36 interaction measured by surface plasmon resonance. The steady-state signal reached at the end of the analyte injection (120 seconds at 30 µl/minute) was plotted against the analyte concentration and the resulting curve fitted with a 1:1 binding model.

Table S1. Bacterial strains used in this study.

**Bacterial strain**	**Source**
Acinetobacter baumannii BCRC80276<br>*S. aureus* BCRC12552 (a.k.a. NCTC8325)	Bioresource Collection and Research Center (Taiwan)
Bacillus cereus ATCC11778<br>*Enterococcus faecium* ATCC35667<br>*Escherichia coli* ATCC25922<br>*Listeria monocytogenes* ATCC19113<br>MRSA ATCC33592<br>*Staphylococcus aureus* ATCC12598<br>*Staphylococcus epidermidis* ATCC12228<br>*Staphylococcus intermedius* ATCC29663<br>*Staphylococcus haemolyticus* ATCC29970<br>*Staphylococcus hominis* ATCC27844<br>*Staphylococcus saprophyticus* ATCC15305<br>*Streptococcus pyogenes* ATCC19615<br>*Streptococcus agalactiae* ATCC13813<br>*Salmonella* Typhimurium ATCC14028	American Type Culture Collection (United States)
Bacillus subtilis 168	Bacillus Genetic Stock Center (United States)
Staphylococcus lugdunensis<br>vancomycin-resistant *Enterococcus faecium*	National Taiwan University Hospital

Table S2. Primers used in this study.

Name	Sequence (5′→3′)
Primers for mutation validation	
CDS789-F	tgttgatcctatcccgcaag
CDS789-R	caaacaaccaaaacactgcga
CDS792-F	aaagcacaacaaggagtgtg
CDS792-R	actgcagcaaattgcaaaattag
CDS936-F	tgcatgttatactagcgatgc
CDS936-R	cgttcagcaagttcttcttgc
CDS2037(YidC2)-F	tccacttattgtttcatacgtactc
CDS2037(YidC2)-R	agcgaaaaataatgtgcgcc
CDS2050-F	ggtagtgttagaaatgatcttgtcca
CDS2050-R	tcgagtatctatttctttaagagaagc
CDS2053-F	aaactgcagatgaatttaatcgcagcagca
CDS2053-R	aaagaattcttatccagcaaatgtcatgaatg
Primers for plasmid construction	
yidC2-P73L-F	tagtttgataacatgaatagtaacaagataacacgaa
yidC2-P73L-R	attcgtgttatcttgttactattcatgttatcaaactat
yidC2-M75A-F	actatttttatagtttgataacgcgaatggtaacaagataa
yidC2-M75A-R	gtgttatcttgttaccattcgcgttatcaaactataaaaatag
yidC2-Y79A-F	gcatcatatgactatttttagcgtttgataacatgaatggta
yidC2-Y79A-R	gttaccattcatgttatcaaacgctaaaaatagtcatatgatg
yidC2-P139L-F	catttgaattaatactagtaaacagcctaatgcactc
yidC2-P139L-R	aagagtgcattaggctgtttactagtattaattcaaatgcc
yidC2-Y188A-F	atacccaagcttggataaaggcaagcacaccggcaataat
yidC2-Y188A-R	tattattgccggtgtgcttgcctttatccaagcttgg
yidC2-L240A-F	agtaagtttggacaattgcgaatgccgcactgaca
yidC2-L240A-R	tctgtcagtgcggcattcgcaattgtccaaacttacttc

Table S3. Mutations identified in the chromosomes of Cpd9-resistant and Cpd36- resistant isolates by whole-genomic resequencing technique.

	Mutation [a]	Locus [b]	Amino acid shift	Location
Cpd9-resistant	A∣G	292840	No change	hypothetical protein
	T∣A	337798	*Intergenic Region*	176 bp at 5' side: possible N-acetyltransferase
	T∣A	2306559	*Intergenic Region*	7 bp at 5' side: 50S ribosomal protein L36
	T∣C	853895	No change	monovalent cation/H^+ antiporter subunit A
	A∣G	906830	*Intergenic Region*	2 bp at 5′ side: regulatory protein Spx
	A∣C	2158671	A184S	membrane protein YidC2 precursor
	G∣A	1720642	No change	universal stress family protein
Cpd36-resistant	A∣G	2158805	P139L	membrane protein YidC2 precursor
	G∣T	2170476	K409N	F_oF_1 ATP synthase subunit alpha
	A∣G	2173080	R39C	F_oF_1 ATP synthase subunit c
	C∣G	28785	G424A	two-component regulator YycH
	C∣A	849570	L410V	putative monovalent cation/H^+ antiporter subunit D
	C∣T	1013496	No change	putative transcriptional regulator
	A∣G	1634860	A150T	luciferase family monooxygenase

[a]The presentation of nucleotide is "Novel / Reference"

[b]The position in the whole-genome sequence of *S. aureus* NCTC8325 (NC_007795).

Table S4. Missense mutations identified in the *yidC2* of Cpd9-, Cpd36- and Cpd46- resistant isolates of *S. aureus.*

	Mutation	Codon change	Amino acid shift
Cpd9-resistant isolate	G550T	GCC→TCC	A184S
Cpd36-resistant isolate	C416T	CCA→CTA	P139L
Cpd46-resistant isolate	C218T	CCA→CTA	P73L

Table S5. The identity and similarity of *S. aureus* YidC2 to the YidC2 and YidC of different Gram-positive and Gram-negative bacteria.

		S. aureus YidC2	
	**Accession number**	**Identity (%)**	**Similarity (%)**
Gram-positive YidC2			
Bacillus cereus	WP_000727738.1	31	50
Bacillus subtilis	AIY95428.1	28	51
Enterococcus faecium	WP_002330077.1	32	53
Listeria monocytogenes	WP_003722505.1	36	58
Streptococcus pyogenes	WP_002985918.1	30	47
Streptococcus agalactiae	WP_000751931.1	30	52
Gram-negative YidC			
Acinetobacter baumannii	WP_001188089.1	12	20
Escherichia coli	WP_000378258.1	13	23
Salmonella Typhimurium	WP_000378281.1	13	23

The alignments of the amino acid sequence of were performed using EMBOSS Needle of EMBL-EBL website.

Table S6. Antimicrobial susceptibilities of *E. coli* wild-type strain ATCC25922, efflux pump deficient (Δ*acrA*) strain JW0452-3, and lipid A deficient (*lpxA2*-ts) strain SM101.

	**MIC (μg/ml)**		
	E. coli **ATCC25922**	***E. coli*** **JW0452-3**[a]	***E. coli*** **SM101**[a]
**Antibiotics**	**37°C**	**37°C**	**28°C**
Cpd36	>64	>64	>64
Cpd46	>64	>64	>64
Erythromycin	64	4	≦0.125
Rifampicin	8	8	≦0.125
Ampicillin	32	16	8
Gentamicin	1	0.5	0.25

[a]The JW0452-3 and SM101 strains of *E. coli* were obtained from the Coli Genetic Stock Center.
